# The Impact of Intracerebral Hemorrhage on the Progression of White Matter Hyperintensity

**DOI:** 10.3389/fnhum.2018.00471

**Published:** 2018-11-28

**Authors:** Xuemei Chen, Xin Chen, Yan Chen, Manman Xu, Tingting Yu, Junrong Li

**Affiliations:** ^1^Department of Neurology, The Affiliated Jiangning Hospital of Nanjing Medical University, Nanjing, China; ^2^Department of Neurology, The Affiliated Drum Tower Hospital of Nanjing Medical University, Nanjing, China

**Keywords:** intracerebral hemorrhage, white matter injury, hematoma, white matter hyperintensity, quantitative analysis

## Abstract

**Objective**: The exact relationship between white matter hyperintensity (WMH) and intracerebral hemorrhage (ICH) after ICH remains unclear. In this retrospective study, we investigated whether patients with ICH had more severe WMH progression.

**Patients and Methods**: A total of 2,951 patients aged ≥40 years with ICH who received brain computed tomography (CT) imaging within 12 h of ICH symptom onset were screened. Ninety patients with two fluid-attenuated inversion recovery (FLAIR) magnetic resonance imaging (MRI) assessments, including 36 patients with Lobar ICH, 40 with basal ganglia region ICH and 14 with ICH at other sites, were included in the final study. We selected 90 age- and gender-matched healthy individuals with two MRI scans as the control group. The WMH volumes at baseline and follow-up were assessed using the FLAIR image by MRICRON and ITK-SNAP software, while the hematoma volumes were calculated based on the CT images using ITK-SNAP software.

**Results**: The annual progression rate of WMH was significantly higher in the ICH group compared with the control group (*p* < 0.05). Furthermore, WMH progression was associated with the ICH volume. The largest ICH volume (>30 mL) was associated with the highest annual progression rate of WMH (*p* < 0.05). In contrast, no trend toward an association between ICH location and the annual progression rate of WMH was observed (*p* > 0.05).

**Conclusions**: Our results showed that ICH patients had more severe WMH progression and that larger ICH volume was related to greater progression of WMH after ICH. These results could provide important prognostic information about patients with ICH.

## Introduction

Intracerebral hemorrhage (ICH) accounts for approximately 10%–15% of all strokes in Western countries and 20%–30% of strokes in Asia and has a high mortality and poor functional outcome (Wen et al., 2018). ICH causes gray matter (GM) destruction as well as proximal or distal white matter injury (WMI) due to complex pathophysiological mechanisms. WM fibers, especially those located within the capsula interna, are one of the most vulnerable tissues in hypertensive ICH. WM hyperintensity (WMH) is a neuroimaging finding of WMI characterized by bilateral, mostly symmetrical, hyperintensities on T2-weighted magnetic resonance imaging (MRI; Wardlaw et al., 2013). Approximately 70% of people over 65 years of age with ICH present with varying degrees of WMH on MRI (de Leeuw et al., 2001; Smith et al., 2017). Increasing WMH volumes are clinically important because they confer risk for developing cognitive impairment, dementia, gait disturbances and depression (Srikanth et al., 2010; Callisaya et al., 2015). Therefore, more attention should be paid to the progression of WMH after ICH.

Previous research has suggested that WMI might reflect the vulnerability of individual brains to pathologic insults and that WMH should be considered when assessing immediate, early, and long-term outcomes after ICH (Zuo et al., 2017). Several examples from the related literature have demonstrated that severe WMH had an impact on larger ICH volumes and hematoma growth; moreover, progression of WMH was an independent predictor of worse functional outcomes in patients with ICH (Lou et al., 2010; Caprio et al., 2013). Our recent research has also shown that greater severity and progression of WMH resulted in larger ICH volumes at baseline (Chen et al., 2018). Therefore, the progression of WMH is an important factor in determining the prognosis of patients with ICH. However, the current studies about WMI and repair after hypertensive ICH are still rare and scattered (Zuo et al., 2017). In recent years, some animal researches have shown that scores of therapeutic agents and methods were effective in the treatment of WMI after hypertensive ICH, although these drugs and treatment methods were rarely used in clinical practice because of the absence of enough clinical studies to support their use (Zuo et al., 2017). A better understanding of the characteristics of WMH following ICH is essential and may shed new light on treatment options. Therefore, in this retrospective study, we aimed to figure out the impact of ICH on the progression of WMH.

## Patients and Methods

### Study Population

**Figure 1:**
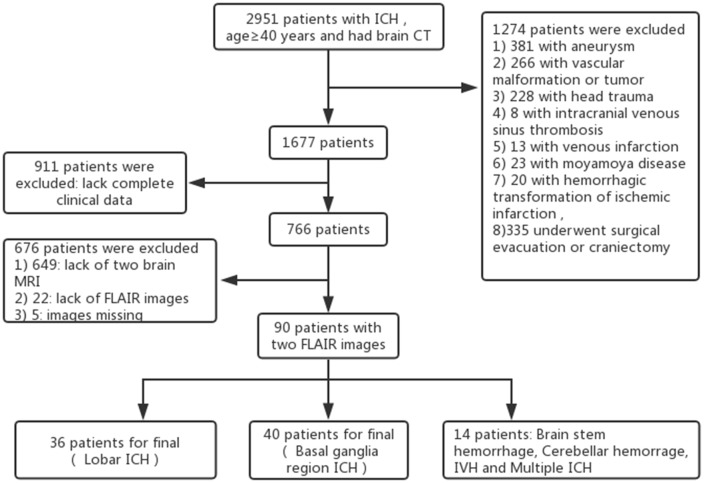
Flowchart of the screening process.

A total of 2,951 patients with ICH were studied retrospectively from January 1, 2012 to November 1, 2017, using data from the Jiangning Hospital imaging center of Nanjing Medical University. The inclusion criteria were as follows: all patients (1) were ≥40 years of age; (2) underwent brain computed tomography (CT) within 12 h of ICH symptom onset; (3) received fluid-attenuated inversion recovery (FLAIR) MRI (the first MRI was performed within 2 days after onset to exclude other disorders of the nervous system and the second MRI was performed at return visit; the interval time between the two scans was 268.36 ± 98.54 days [mean ± stand deviation (SD)], range from 3 months to 3 years); and (4) had baseline clinical and demographic information available, including demographic characteristics, medical history, physical examination results and laboratory examination findings. The exclusion criteria included having an underlying aneurysm, vascular malformation or tumor, head trauma, venous infarction, Moyamoya disease, hemorrhagic transformation of ischemic infarction and previous surgical evacuation or craniectomy. We selected 90 age- and gender-matched healthy individuals who underwent regular health check at Jiangning Hospital with two MRI scans as the control group (the interval time between the two scans was 300.47.36 ± 96.06 days [mean ± SD], range from 3 months to 3 years). The exclusion criteria for healthy individuals were as follows: (1) parenchymal hemorrhage or subarachnoid hemorrhage; (2) history of ischemic stroke or cardiogenic cerebral infarction; (3) other neurological diseases (e.g., Parkinson’s disease, epilepsy, AD, multiple sclerosis and neuromyelitis optica); (4) systemic disease (cancer, shock, anemia, or thyroid dysfunction); (5) psychiatric disease; and (6) MRI technical difficulties (Figure 1).

The Ethics Committee of Jiangning Hospital, affiliated with Nanjing Medical University, approved this study. The study procedures were conducted according to institutional guidelines.

### Risk Factors

To assess relevant risk factors at baseline, we collected data on patients’ clinical and demographic characteristics, including age and sex; comorbid conditions, including history of hypertension, history of diabetes mellitus, presence of coronary heart disease, history of dyslipidemia and history of stroke or transient ischemic attack (TIA); medications used on admission, such as antiplatelet agents, anticoagulants and statins; systolic and diastolic blood pressure (SBP and DBP) on initial evaluation; past or current cigarette or alcohol use; and laboratory results, including tests for glucose [fasting blood glucose (FBG) and glycosylated hemoglobin (HbAIc)], total cholesterol (TC), high-density lipoprotein (HDL), triglycerides (TG), low-density lipoprotein (LDL), Apolipoprotein A-I (apoA I), C-reactive protein (CRP), blood urea nitrogen (BUN), creatinine (Cr) and uric acid (UA).

### Radiologic Data

#### CT Acquisition and Analysis

**Figure 2:**
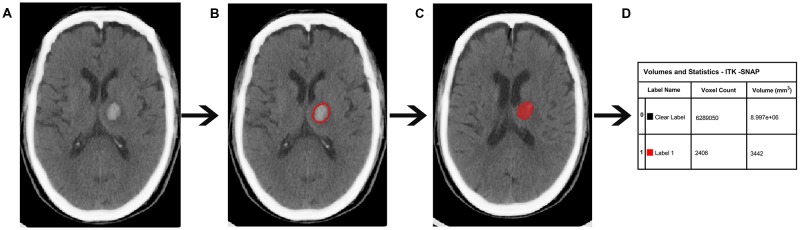
Quantitative measurement of intracerebral hemorrhage (ICH) volume. **(A)** Original computed tomography (CT) image. **(B)** The high-density area. **(C)** A sketch of the high-density area. **(D)** Calculation of the ICH volume.

CT scanning was performed on a Lightspeed scanner (GE Medical system) and images were obtained with the following parameters: acquisition matrix = 512 × 512, thickness = 10.0 mm. At baseline, the hematoma volumes of the 90 ICH patients were measured with ITK-SNAP software (University of Pennsylvania, Philadelphia, PA, USA)^1^. The baseline scan was limited to 12 h because most of the hematoma expansion occurred within the first 24 h following ICH onset (Figure 2).

#### MRI Acquisition and Analysis

MRI scanning was performed on a 3.0-T scanner (Achieva 3.0 T Ingenia; Philips Medical Systems, Eindhoven, Netherlands) with a 32-channel head coil. High-resolution T1-weighted axial images covering the whole brain were obtained by a 3D-magnetization prepared rapid gradient-echo sequence: TR = 9.8 ms; FA = 8°; TE = 4.6 ms; FOV = 256 × 256 mm; acquisition matrix = 256 × 256; gap = 0 mm, thickness = 1.0 mm; number of slices = 192. Additionally, the T2 FLAIR axial images were obtained with the following parameters: TR = 4,500 ms; TE = 344 ms; FA = 90°; acquisition matrix = 272 × 272; thickness = 1 mm; gap = 0 mm, number of slices = 200.

#### Radiological Diagnosis of WMH

WMH is defined as hyperintense on T2-weighted or FLAIR sequences but can appear as isointense or hypointense (although less hypointense than cerebrospinal fluid, CSF) on T1-weighted sequences, depending on the sequence parameters and the severity of the pathological changes. WM lesions, characterized by bilateral and mostly symmetrical hyperintensity on T2-weighted or FLAIR sequences, are common in older individuals.

#### Quantitative Analysis of WMH Volume

**Figure 3:**
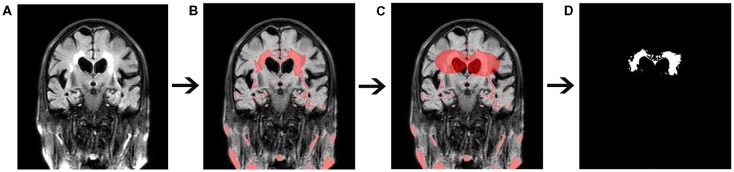
Quantitative measurement of white matter hyperintensity (WMH) volume. **(A)** Original fluid-attenuated inversion recovery (FLAIR) image. **(B)** The high-signal area. **(C)** A sketch of the affected WMH area. **(D)** Extraction of the affected WMH area.

WMH volumes were quantified using FLAIR imaging by the software programs MRICRON (University of Nottingham School of Psychology, Nottingham, UK)^2^ and ITK-SNAP (Chen et al., 2006; Rost et al., 2010; Figure 3). All scans were checked by visual inspection. First, the MRICRON software was used to extract the effective WMH area (the first step was to import original FLAIR image, the second step was to extract the high-signal area, the third step was to sketch the contours of the affected WMH area, and the fourth step was to reconstruct the shape of WMH). Ultimately, the ITK-SNAP software was used to calculate the WMH volume.

CT and MRI scans were reviewed independently by two investigators. The test-retest intraclass correlation coefficients for inter- and intra-observer agreements were >0.90.

#### Statistical Analysis

Mean (SD) or median (interquartile range) values were used to describe continuous variables, while frequencies and percentages were used for categorical variables. Normality was determined by the Kolmogorov-Smirnov test. To compare the categorical variables of clinical features, chi-square tests were used, and the independent *t*-test was used to compare continuous variables with normal distribution while the Mann-Whitney *U-test* was used to compare continuous variables with non-normal distribution. The annual progression rate of WMH was calculated by subtracting the baseline WMH volume from the follow-up WMH volume on MRI scans, dividing this value by the baseline WMH volume, and finally dividing the result by year. Univariate linear regression analysis was carried out to identify potential risk factors for the annual progression rate of WMH. Univariate predictors at a level of *p* < 0.10 were considered significant and were entered into a multivariate linear regression model. To determine the effect of ICH volume on the annual progression rate of WMH, ICH patients were categorized into quartiles according to the baseline ICH volume measured on CT. Intergroup differences were analyzed by one-way ANOVA followed by Bonferroni *post hoc* test. All tests were two-tailed; *p* < 0.05 was considered statistically significant. All statistical analyses were performed using Statistical Product and Service Solutions (SPSS) version 16.0 (SPSS Inc., Chicago, IL, USA).

## Results

### Baseline Characteristics

As shown in Table 1, differences in the demographic characteristics between the ICH and control group did not reach statistical significance (*p* > 0.05). Moreover, there were no significant differences in past medical history, medications, smoking habit, alcohol intake, or laboratory examinations between the ICH and control groups (*p* > 0.05).

**Table 1:** Baseline characteristics.

Item	ICH (*n* = 90)	Control (*n* = 90)	*P* value
Age, years, mean ± SD	66.68 ± 13.38	69.56 ± 10.62	0.11
Men, *n* (%)	59 (66%)	57 (63%)	0.76
Past medical history, *n* (%)
Hypertension	48 (53%)	50 (56%)	0.09
Coronary artery disease	17 (19%)	25 (28%)	0.09
Diabetes mellitus	14 (16%)	27 (30%)	0.56
Hyperlipidemia	2 (2%)	6 (7%)	0.27
TIA or storke	19 (21%)	22 (28%)	0.70
Smoking, *n* (%)	15 (17%)	18 (20%)	0.82
Alcohol, *n* (%)	10 (17%)	15 (17%)	0.68
Medications, *n* (%)
Use of antiplatelet agents	19 (21%)	24 (27%)	0.95
Use of anticoagulation	5 (6%)	4 (7%)	0.46
Use of statin	13 (14%)	19 (21%)	0.69
Clinical variables, mean ± SD
SBP, mm Hg	144.37 ± 20.38	138.89 ± 16.30	0.07
DBP, mm Hg	78.28 ± 10.52	75.98 ± 8.52	0.14
FBG, mM	5.80 ± 1.38	5.49 ± 1.67	0.26
HbAIc, %	6.06 ± 0.51	6.46 ± 1.39	0.77
TC, mM	4.16 ± 1.35	4.09 ± 0.99	0.74
TG, mM	1.48 ± 0.71	1.34 ± 0.80	0.21
LDL, mM	2.20 ± 0.92	2.22 ± 0.74	0.90
HDL, mM	1.09 ± 0.38	1.11 ± 0.35	0.76
ApoA I, g/L	1.12 ± 0.40	1.13 ± 0.35	0.94
BUN, mM	5.90 ± 2.50	6.23 ± 3.18	0.51
Cr, μM	90.57 ± 86.73	90.29 ± 104.10	0.99
UA, μM	311.15 ± 109.71	331.22 ± 96.24	0.26
CRP, mg/L	15.96 ± 34.78	14.28 ± 27.34	0.75

*Note: values with normal distribution are presented as the mean ± stand deviation (SD). Abbreviations: ICH, intracerebral hemorrhage; TIA, transient ischemic attack; SBP, systolic blood pressure; DBP, diastolic blood pressure; FBG, fasting blood glucose; HbAIc, glycosylated hemoglobin; TC, total cholesterol; TG, triglycerides; HDL, high-density lipoprotein; LDL, low-density lipoprotein; ApoA I, Apolipoprotein A-I; CRP, C-reactive protein; BUN, blood urea nitrogen; Cr, creatinine; UA, uric acid*.

### Analysis of the Annual Progression Rate of WMH Between the ICH and Control Groups

A comparison of the annual progression rates of WMH between the ICH and control groups is shown in Table 2. The volume of WMH increased by 20% annually in the control group; however, the annual progression rate of ICH patients increased by 33%, which was significantly higher than that of control patients (ICH vs. control: 0.33 ± 0.41 vs. 0.20 ± 0.32, *p* = 0.01).

**Table 2:** Annual progression rate of WMH between ICH and control group.

	ICH	Control	*P* value
Annual progression rate of WMH, %, mean ± SD	0.33 ± 0.41	0.20 ± 0.32	0.01*

Note: *ICH vs. control significant difference (*P* < 0.05). Abbreviations: ICH, intracerebral hemorrhage; WMH, white matter hyperintensity.

### Univariate and Multivariate Linear Regression of Risk Factors for the Annual Progression Rate of WMH

We next determined the association between the annual progression rate of WMH and risk factors using linear regression analysis. The univariate linear regression analysis showed that ICH, hypertension history, TIA or stroke history, SBP and DBP were risk factors for the annual progression rate of WMH. However, multivariate linear regression analysis showed that only ICH and TIA or stroke history were independently associated with the progression rate of WMH after adjusting for other confounding variables (*p* < 0.05; Table 3).

**Table 3:** Univariate and multivariate linear regression of risk factors of annual progression rate of WMH.

	Univariate		Multivariate	
	β	*P* value	β	*P* value
ICH	−0.14	0.01*	−0.17	0.02*
Hypertension history	0.14	0.03*	-	-^#^
TIA or stroke history	−0.12	0.08	0.14	0.04*
SBP	0.00	0.03*	0.00	0.34
DBP	0.01	0.02*	0.01	0.14

Note: *significant difference (P < 0.05), ^#^since the history of hypertension, systolic and diastolic pressure was not independent, in the multiple regression model, only SBP and DBP were included. Abbreviations: ICH, intracerebral hemorrhage; WMH, white matter hyperintensity; TIA, transient ischemic attack; SBP, systolic blood pressure; DBP, diastolic blood pressure.

### Linear Regression of the Association of ICH Volume and ICH Position With the Annual Progression Rate of WMH

In univariate linear regression analysis, increasing ICH volume was a positive predictor of the annual progression rate of WMH (*p* < 0.05); specifically, a higher annual progression rate of WMH was associated with a larger ICH volume at baseline (*p* = 0.03). Nevertheless, no trend toward an association between ICH position and the annual progression rate of WMH was observed (*p* = 0.20; Table 4).

**Table 4:** Univariate linear regression of ICH volume and ICH position of annual progression rate of WMH.

	β	*P* value
ICH volume	8.987E-6	0.03*
ICH position	0.08	0.20

Note: *significant difference (P < 0.05). Abbreviations: ICH, intracerebral hemorrhage; WMH, white matter hyperintensity.

### Analysis of the Relationship of ICH Volume and the Annual Progression Rate of WMH According to Quartiles of ICH Volume

**Figure 4:**
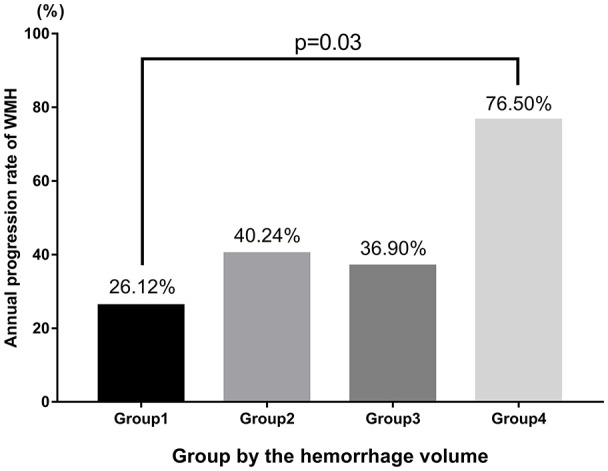
Analysis of the relationship between ICH volume and the annual progression rate of WMH according to quartiles of ICH volume.

In addition, we further assessed the link between the effect of ICH volume and the annual progression rate of WMH. We categorized patients into four groups according to quartiles of ICH volume: group 1 (0%–25%: <10 mL), group 2 (26%–50%: 10–20 mL), group 3 (51%–75%: 21–30 mL) and group 4 (76%–100%: >30 mL). We found that patients in group 4 showed a 76% annually progressive rate, which is significantly higher than that in group 1 (group1 vs. group 4: 0.26 ± 0.37 vs. 0.76 ± 0.45, *p* = 0.03; Table 5, Figure 4).

**Table 5:** Analysis of relationship of ICH Volume and annual progression rate of WMH according to quartered ICH Volume.

	Hemorrhage volume				
	1	2	3	4	*P*
	0–25% (*n* = 57)	2–50% (*n* = 17)	51–75% (*n* = 10)	76–100% (*n* = 6)	value
Hemorrhage volume (mm^3^)	3881.93 ± 2498.65	13498.71 ± 2739.28	24795.00 ± 3161.74	36506.67 ± 4249.57	-
Annual progression rate of WMH (%)	0.26 ± 0.37	0.40 ± 0.29	0.37 ± 0.71	0.76 ± 0.45	0.02*

Note: values are presented as the mean ± SD; *intergroup differences (P = 0.02), 1 vs. 4 significant difference (P = 0.03). Abbreviations: ICH, intracerebral hemorrhage; WMH, white matter hyperintensity.

## Discussion

The main findings of this study were as follows: (1) ICH patients had more severe WMH progression after ICH than patients without ICH, according to quantitative WMH measurements; and (2) WMH progression was associated with the ICH volume at baseline, larger ICH volume and higher annual progression rate of WMH.

It is generally known that WMH is a highly prevalent disease in older individuals. The presence and severity of WMH may be linked to the cumulative effects of multiple factors such as age, hypertension, cerebral infarction, lacunar infarction, history of ICH and increased TG (Zhang and Kang, 2013; Xu et al., 2014; Bivard et al., 2016). Consistent with previous studies, we also found that ICH, TIA or stroke history, SBP and DBP were associated with the progression of WMH in our study population. Among these risk factors, a history of ICH was recognized as the most important vascular contributor because it was an independent predictor of the annual progression rate of WMH. ICH and LA share several risk factors in common (hypertension, cerebrovascular disease), and may share a common underlying pathological mechanism involving microangiopathy (Caprio et al., 2013). Interestingly, the relationship between ICH and WMH progression is a mutually promoting process, not merely a unilateral process. Recent studies have shown that severe WMH was associated with spontaneous lobar hemorrhage and a greater risk of local hemorrhage after intravenous thrombolysis (IVT; Chen et al., 2018). In addition, more extensive WM damage was detected in patients with ICH than in those with ischemic stroke or other cerebral small vessel pathologies (Rost et al., 2010).

Most single hemorrhages were found in deep (subcortical) sites, including the basal ganglia (34.2%), thalamus (8.3%), cerebellum (6.8%), ventricles (1.5%) and brainstem (1.1%; Hu et al., 2013). However, we do not know whether the ICH position affects the progression rate of WMH. One previous publication indicated that the WMH volume was similar in deep hemispheric ICH and lobar ICH and did not find major differences in supratentorial WMH distribution (Smith et al., 2017). In this study, we also did not find a significant association between the annual progression rate of WMH and ICH position, which was consistent with the results of Smith et al. (2010). Increasing research on WMI after hypertensive ICH should focus on these sites in the future.

Compared to ischemic stroke, ICH has higher mortality and leads to more severe disability (Keep et al., 2012). Known poor prognostic factors of ICH include large hematoma volume, hematoma expansion, intraventricular hemorrhage, infratentorial location, older age and anticoagulation treatment (An et al., 2017); hence, ICH volume is an important factor in determining the prognosis of ICH patients. A previous study found that a hematoma volume exceeding approximately 25 mL was a precondition for the occurrence of WM damage (Sugimoto et al., 1989). A clear link between the progression of WMH and volume of ICH was observed in our study. The progression of WMH was volume-dependent, and the group with the highest ICH volume had a significantly higher annual progression rate of WMH. Massive ICH volume can trigger more serious WM damage.

ICH is usually caused by ruptured vessels that are degenerated due to long-standing hypertension. Cerebral autoregulation (CA) is a mechanism which protects brain tissue from either hyperperfusion or hypoperfusion within a wide range of BP fluctuations (Ma et al., 2016). Dysfunction of automatic regulation could be the prime contributor to the occurrence of ICH. Notably, WMH is also attributed to impairment of cerebral hemeodynamics, because the development and progression of WMH occur, in part, as a result of regional hypoperfusion of susceptible normal-appearing WM (Etherton et al., 2016). Therefore, WMH and ICH can have a common pathological pathway (Zuo et al., 2017). Furthermore, ICH could exacerbate demyelination and downregulation of myelin basic protein (MBP) expression in the WM. Many pathological molecules such as TNF-α and cytokines were highly expressed or overactivated. These pathological molecules had aggravated the damage to myelin sheaths and then affected the morphology and function of the WM (Zuo et al., 2017). Increasing research has suggested that WMI will seriously influence the prognosis of hypertensive ICH patients (Callisaya et al., 2015). Severe WMI may reflect the vulnerability of the brain to further insults and predicts poor outcome after ICH (Lee et al., 2010; Tveiten et al., 2013; Lambert et al., 2016; Yamashita et al., 2016). In summary, WMI after hypertensive ICH was caused by a variety of pathogenic factors and resulted in a worse long-term prognosis. Further research is needed to explore the exact pathogenic mechanisms for WMI after hypertensive ICH.

However, this study has some limitations. First, although the records of quantities of patients were screened, only 90 patients met the inclusion criteria, which decreased the statistical power of this study. Second, although the interval between two MRI examinations is usually around 1 year, the accuracy of the annual progression rate of WMH may be influenced by the large range of follow up time (3 months to 3 years). Finally, WMH is a reaction to a serious WMI; thus, we may ignore the disruption of normal-appearing WM. Recently, diffusion tensor imaging (DTI) was shown to be the most suitable for detecting microstructural abnormalities in WM at the early stages (Jang et al., 2013; Goh et al., 2015). To better understand the damage to WM fibers, research using DTI should be the top priority in the future (Tamura and Araki, 2015).

## Conclusion

In conclusion, our study was the first to use quantitative analysis to show that ICH could trigger more severe WMH, and that a larger ICH volume was associated with a greater progression of WMH after ICH. Consequently, our findings suggested that the progression of WMH might provide important prognostic information about patients with ICH and could be proposed as a clinically relevant disease marker. Thus, it is important to control the risk factors to reduce the occurrence of ICH and improve the functional outcomes of patients after ICH. Additionally, the study confirms the importance of pharmacologically modulating WMH volume at the early stages of WMH development. Further studies should be undertaken to elucidate the pathophysiologic link between WMH and ICH and to confirm our findings.

## Author Contributions

All authors had full access to all the data in the study and take responsibility of the data and the accuracy of the data analysis. XuemeiC: study concept and design. XuemeiC, XinC and MX: acquisition of data. YC and XuemeiC: statistical analysis. XuemeiC and XinC: visualization. XuemeiC, XinC, YC, MX, TY and JL: writing—review and editing.

## Conflict of Interest Statement

The authors declare that the research was conducted in the absence of any commercial or financial relationships that could be construed as a potential conflict of interest.

## Footnotes

^1^www.itksnap.org

^2^www.mricro.com

## Abbreviations

apoA I: apolipoprotein A-I
BUN: blood urea nitrogen
Cr: creatinine
CRP: C-reactive protein
CSF: cerebrospinal fluid
CT: computed tomography
DBPs: diastolic blood pressures
DTI: diffusion tensor imaging
FBG: fasting blood glucose
FLAIR: fluid-attenuated inversion recovery
GM: gray matter
HbAIc: glycosylated hemoglobin
HDL: high-density lipoprotein
ICH: intracerebral hemorrhage
IVT: intravenous thrombolysis
LDL: low-density lipoprotein
MBP: myelin basic protein
MRI: magnetic resonance imaging
SBPs: systolic blood pressures
TC: total cholesterol
TG: triglycerides
TIA: transient ischemic attack
UA: uric acid
WM: white matter
WMH: white matter hyperintensity
WMI: white matter injury.
